# Verification of fCAL turbo Buhlmann reagent for determining serum calprotectin

**DOI:** 10.1515/almed-2024-0201

**Published:** 2025-06-30

**Authors:** Helena Čičak, Željka Šarčević, Ana Sruk, Frane Bukvić, Daria Pašalić, Lora Dukić, Ana-Maria Šimundić

**Affiliations:** Department of Medical Laboratory Diagnostics, University Hospital “Sveti Duh”, Zagreb, Croatia; Department of Neurology, University Hospital “Sveti Duh”, Zagreb, Croatia; Department of Orthopaedic Surgery and Traumatology, University Hospital “Sveti Duh”, Zagreb, Croatia; Department of Medical Chemistry, Biochemistry and Clinical Chemistry, University of Zagreb School of Medicine, Zagreb, Croatia; Faculty of Pharmacy and Biochemistry, University of Zagreb, Zagreb, Croatia

**Keywords:** calprotectin, preanalytical, serum, verification

## Abstract

**Objectives:**

The aim of this study was to verify Buhlmann fCAL turbo reagent, used for analysing calprotectin in stool, on serum samples.

**Methods:**

Leftover patients’ serum samples were used. Verification protocol included: precision performed in accordance with Clinical and Laboratory Standards Institute (CLSI) EP15-A3 guidelines; comparison of Buhlmann fCAL turbo and serum GCAL Gentian reagents; verification of reference intervals (RI) proposed from literature (0.68–5.45 mg/L) in accordance with CLSI EP28-A3C guidelines; limit of blank (LoB) and limit of quantitation (LoQ) performed in accordance with CLSI EP17-A2 guidelines; linearity performed in accordance with CLSI EP6-A guidelines; carry-over and lastly, hook effect. Acceptance criteria were based on manufacturer specifications stated in the package insert for the fCAL turbo Buhlmann reagent designed for analysing calprotectin in stool samples.

**Results:**

The CVs% for precision were within acceptance criteria, ≤10 %. Bland-Altman analysis showed the presence of a bias of 3.4 mg/L (95 % confidence interval 2.00–4.79). The equation for the Passing-Bablok regression was y= −0.01 (−0.08 to 0.06) + 0.37 (0.36–0.40)x, and it showed proportional difference. The obtained LoB was 0 mg/L, while the LoQ was 0.04 mg/L (CV% ≤ 20 %). The method was linear in the range 0.04–4.14 mg/L, and carry-over (0.1 %) and hook effect were not detected. The proposed RI in the literature was verified using 20 leftover patients’ samples.

**Conclusions:**

Despite being declared for analysing stool samples only, Buhlmann fCAL turbo reagent can be used to determine serum calprotectin. Buhlmann fCAL turbo reagent is not comparable to the GCAL Gentian reagent.

## Introduction

Serum calprotectin in literature is usually mentioned as a promising biomarker for various diseases from different disciplines of medicine, e.g., gastroenterology, orthopaedics, neurology, and many more. Most of the calprotectin concentration is in the cells, mainly neutrophils. Neutrophils are the first line of defence against pathogens, and they are the most numerous of all leukocytes in blood. Thus, during the first immune response, calprotectin is released from neutrophils and other immune cells into the environment where the immune reaction occurs, usually in the surrounding tissue or body fluids (BF) [1].

Determination of calprotectin concentration in BFs, e.g., synovial fluid, has great potential for learning clinically relevant information about the local inflammation rate. However, BF sampling is not always standardized and sometimes cannot be repeated if the sample volume is insufficient. Additionally, on some occasions, the BF sampling has a risk of whole blood interference and contamination because of the traumatic punction. In case of trauma, the measured concentration of calprotectin from BF is not appropriate, not because of the blood calprotectins’ significant contribution to the concentration, but because the exact contribution is not known or cannot be estimated. Also, in certain cases, monitoring calprotectin concentration dynamic would have a significance to the clinical decision-making process for the patient. Unfortunately, the repetitive sampling of BFs is infrequent due to the invasive procedure and many other risks of potential complications caused by the sampling process.

On the other hand, venous blood sampling is standardized and, in most cases, can be repeated as many times as necessary. Also, serum or plasma samples are more accessible. Thus, there is a lack of information in literature about the verification of the fCAL turbo Buhlmann reagent, widely used for faecal samples, for serum calprotectin, e.g., the verification of the limit of quantitation.

Until recently, only ELISA kits and reagents for automated analysers for the determination of faecal calprotectin were available, following its clinical use and introduction into the medical laboratory routine. In recent years, serum calprotectin reagents (ready-to-use) have been developed, focusing on the determination on automated analysers. Only few publications performed verification of serum or plasma calprotectin by using fCAL turbo (Buhlmann Laboratories AG, Schonenbuch, Switzerland), GCAL – Gentian Serum Calprotectin (Gentian Diagnostics, Moss, Norway), DiaSorin Liaison Calprotectin assay (DiaSorin, Saluggia, Italy), and Phadia 250 EliA Calprotectin flouroenzymeimmunoassay [2], [3], [4], [5]. To the best of our knowledge, there is no information about extensive validation or verification of reagents for calprotectin on serum samples.

Our hypothesis is that the reagent for the determination of faecal calprotectin would also be suitable for determining the concentration of calprotectin in serum samples.

The aim of this study was to verify the Buhlmann fCAL turbo reagent on serum samples by estimating precision, comparing the two reagents – fCAL turbo Buhlmann and GCAL Gentian, verifying reference intervals proposed in literature, establishing the limit of the blank, limit of quantitation, linearity, carryover, and hook effect.

## Materials and methods

The study was performed at the University Hospital “Sveti Duh”, in Zagreb, Croatia. This study used the leftover patients’ serum samples after the physicians’ ordered tests were obtained. The samples were collected into a 4 mL laboratory collection tube with a clot activator (REF 11010) (Vacutest Kima s.r.l., Arzergrande, Italy). The venous blood sampling was done using the European Federation of Clinical Chemistry and Laboratory Medicine (EFLM) and the Latin America Confederation of Clinical Biochemistry (COLABIOCLI) recommendations [6]. After blood sampling and transporting them to the laboratory, the samples were centrifuged in a VWR MEGA STAR 1.6 centrifuge (VWR International GmbH, Vienna, Austria) at 4,000 rpm for 10 min. After centrifugation, the ordered tests were finished within 2 h from sampling. Immediately after, the serum samples were used to conduct the procedures in this study. The Buhlmann fCAL turbo reagent (Buhlmann Laboratories AG, Schonenbuch, Switzerland) was used to analyse calprotectin concentrations. The reagent was applied to the analyser Atellica Solution (Siemens, Erlangen, Germany) with the recommended application settings defined by the manufacturer. This reagent is intended for measuring the calprotectin in stool. Prior to analysis, faecal samples were diluted with extraction buffer in ratio 1:500. Serum samples don’t need extraction and dilution for analysis. Because of the dilution, all the obtained results were divided by 500. Because of this, all our verification aims and objectives produced by the manufacturer and results from the analyser were displayed in µg/g with divided concentrations in brackets. Furthermore, the manufacturer states that the results could be converted from µg/g to mg/L by applying a factor of 1.0. Both used reagents (fCAL turbo Buhlmann and Gentian) were based on the same methodology which is immunoturbidimetry. The sample is mixed and with immunoparticles which are coated with calprotectin-specifc antibodies. The calprotectin in the sample reacts with immunoparticles and the agglutination increases the turbidity which is measured by light absorbance and it is proportional to concentration of calprotectin in the sample. The calibrators and controls used in this study for fCAL turbo Buhlmann were B-KCAL-CONSET (lot 3616) and B-KCA-CASET (lot 3616), and for Gentian reagent was GCAL calibrator kit (lot 2007406) and GCAL control kit (2007409), respectively. The study was done with the approval of the hospital Ethics Committee for using leftover blood samples.

### Verification of precision

For estimating precision, two patients’ samples were used. The precision study was performed following CLSI EP15-A3 guidelines [7]. Two samples with different calprotectin concentrations (high (H) and low (L)) were analysed five times in five consecutive days. The samples were aliquoted in 5 aliquots and stored at −20 °C until the analysis. The acceptance criterion was declared in the Buhlmann fCAL turbo package insert, and it was CV% ≤ 10 %.

### Method comparison

Two different reagents analysed 40 patients’ serum samples: a) Buhlmann fCAL turbo and b) GCAL – Gentian Serum Calprotectin (Gentian Diagnostics, Moss, Norway). All samples were measured in a single run and were analysed on a Siemens Atellica Solution analyser. The results obtained by the Buhlmann fCAL turbo reagent were divided by 500, as explained before. Gentian results do not need any factor conversion.

### Verification of the limit of the blank and the limit of quantitation

The limit of blank (LoB) and limit of quantitation (LoQ) were estimated according to the CLSI EP17-A2 guidelines [8]. Two saline solution samples were analysed for three consecutive days in the following replicates per day: 3-3-4 (20 replicates in total). The declared LoB by Buhlmann was 16.7 μg/g (0.03 mg/L). The acceptance criteria for LoB verification is 85 %, i.e., 17 out of 20 results should be equal to or lower than the LoB declared by the manufacturer.

For estimating LoQ, patients’ samples with low concentrations of calprotectin were used. The samples’ dilution was performed with CH diluent (Siemens, Erlangen, Germany), recommended by the manufacturer, in order to have low calprotectin concentrations (nearing declared LoQ). The samples were analysed for three consecutive days in replicates 3-3-4 per day (20 replicates in total). The manufacturer’s declared LoQ is 20 μg/g (0.04 mg/L), according to the application documents for Siemens Atellica Solution, but in the general package insert LoQ was declared as 23.7 μg/g (0.05 mg/L) [9], 10]. We decided to verify the LoQ declared for our specific analyser used in this study. The acceptance criteria for verifying LoQ was CV% ≤ 20 %.

### Verification of linearity

Declared linearity was 20–2000 μg/g (0.04–4.00 mg/L). The linearity was performed using CLSI EP6-A guidelines [11]. Two patients’ samples with high (H) and low (L) calprotectin concentrations were used, targeting the upper and lower ends of declared linearity, respectively. The sample with a high calprotectin concentration was obtained by spiking it with human recombinant calprotectin (ref. no. HC2120, HycultBiotech Inc., Wayne, USA). The serial dilutions were made, and the six concentration ranges were made by mixing L and H samples in predefined volume ratios as follows: 1) L, 2) 0.8L + 0.2H, 3) 0.6L + 0.4H, 4) 0.4L + 0.6H, 5) 0.2L + 0.8H, and 6) H. All samples were analysed once. The results were compared to the expected values, which were calculated after measuring H and L concentrations. Acceptance criteria for maximum deviation were less than 10 %, as declared by the manufacturer [7].

### Verification of carry-over

Two serum samples, with high (H) and low (L) calprotectin concentrations, were divided into 4 aliquots, each. The aliquots were analysed in the following order: H1, H2, H3, H4, L1, L2, L3, and L4. The carryover was calculated according to this formula: {L1 − (L3 + L4)/2}/{(H3 + H2)/2 − (L3 + L4)/2}*100, and was considered acceptable if it was <1 % [12].

### Hook effect

Manufacturer does not report any existence of hook effect. The appointed sample was spiked with human recombinant calprotectin (ref. no. HC2120, HycultBiotech Inc., Wayne, USA). After measuring its’ calprotectin concentration, 9 diluted samples were produced by using serial dilution with CH diluent. If the concentration in diluted samples were higher than in the initial spiked sample, it would be considered that the hook effect was detected [3].

### Verification of reference intervals (RI)

According to CLSI EP28-A3C guidelines, verifying the reference interval of calprotectin must include 20 leftover serum samples from outpatients who came for general medical examination [13] In this part of the study, all patients without any previous or current medical conditions were included. Exclusion criteria were: a) presence of acute or chronic inflammatory conditions or diseases (CRP, sedimentation of erythrocytes, and the absolute number of leukocytes had to be within the reference intervals); b) patient age ≤18 and ≥65 years old; and c) haemolysed or lipemic samples. The manufacturer does not declare reference intervals for serum samples. Literature data declares a reference interval of 0.68–5.45 mg/L for the concentration of calprotectin obtained by the Buhlmann fCAL turbo reagent in serum samples [2]. Acceptance criteria for verifying RI is 18/20 obtained results within the proposed RI.

### Statistical analysis

For the statistical analysis of the obtained data, Microsoft 365 Excel (Microsoft, Washington, USA) was used, and the data were described by a descriptive statistics. All data that are not normally distributed were shown as a median and interquartile range (IQR). Also, the method comparison was statistically analysed using Bland-Altman analysis and Passing-Bablok regression using the statistical software MedCalc 20.027 (Ostend, Belgium). p<0.05 was considered as the level of significance.

## Results

### Verification of precision

The concentrations of two patients’ samples that were used for precision verification were 2.29 mg/L for sample 1 and 3.94 mg/L for sample 2. The calculated CV% (coefficient of variation) for both samples, for within-run, between-run, and within-laboratory were 2.22 %, 6.73 %, and 7.09 % for sample 1, and 1.19 %, 8.16 %, and 8.25 % for sample 2, respectively. All CV%s were within the acceptance criteria.

### Method comparison

**Figure 1:**
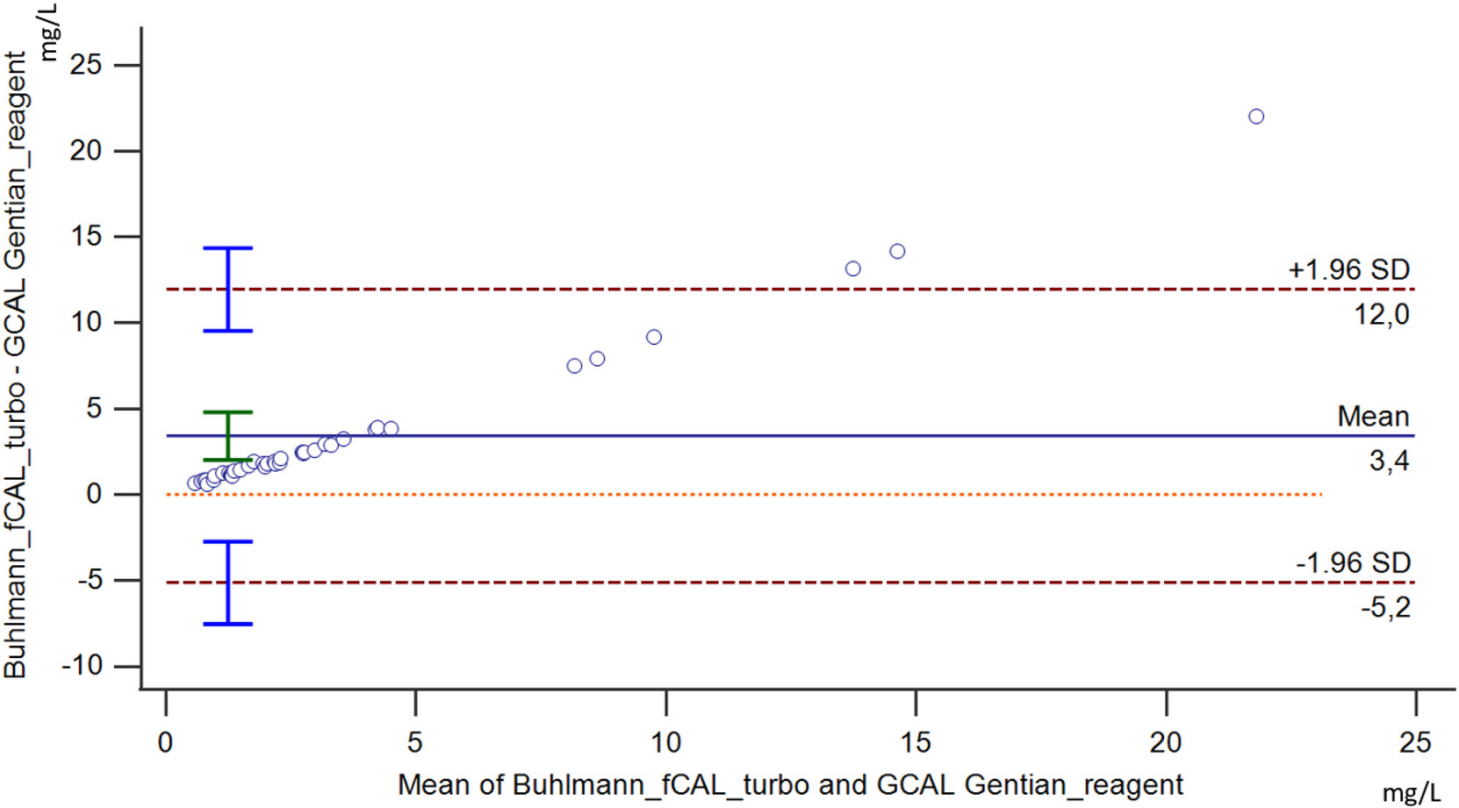
Bland-Altman plot for method comparison shows absolute bias for comparison of the Buhlmann fCAL turbo and GCAL – Gentian Serum Calprotectin. The blue solid line represents absolute bias with a 95 % confidence interval (Cl) as a green error bar.

**Figure 2:**
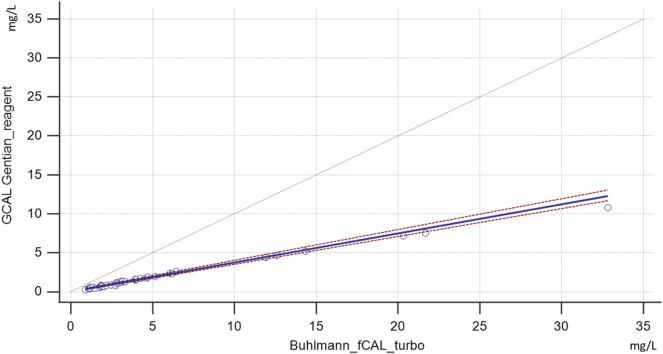
Passing-Bablok regression of two measurements by reagents Buhlmann fCAL turbo and GCAL – Gentian Serum Calprotectin. On the x-axis are the measurements obtained by Buhlmann reagent, and on the y-axis are the measurements obtained by Gentian reagent. The blue solid line is a regression line with 95 %Cl, which is shown as red dashed lines.

The method comparison for serum calprotectin is shown in Figures 1 and 2. Bland-Altman analysis showed positive constant bias of 3.4 (95 % Cl 2.00–4.79) and positive proportional bias of 92.8 % (95 % Cl 89.95 %–95.56 %). The cusum test from the Passing-Bablok regression showed no significant deviation from linearity (p=0.150). Also, the regression equation was y= −0.01 (−0.08 to 0.06) + 0.37 (0.36–0.40)x, and it showed a proportional difference.

### Verification of the limit of the blank and the limit of quantitation

The 20 out of 20 results from analysing two saline samples were lower than the LoB declared by the manufacturer. The LoB of 16.7 μg/g (0.03 mg/L) was verified. Additionally, all results from analysing saline were 0 mg/L.

By analysing two serum samples from two individual patients, the mean concentrations were 20.5 μg/g (0.04 mg/L) and 22.1 (0.04 mg/L) µg/g, and the CV%s obtained were 13.7 % and 12.1 %, respectively. CV%s were within acceptance criteria (≤20 %), and the declared LoQ by the manufacturer (20.0 μg/g (0.04 mg/L)) was verified.

### Verification of linearity

The calprotectin concentrations of low and high samples were 20.82 μg/g (0.04 mg/L) and 2070.21 μg/g (4.14 mg/L). The expected concentrations of predefined dilutions were calculated. The biases from expected and measured values were less than 10 %, except for dilution 0.8L + 0.2H, whose bias was 10.5 %. The linearity data are presented in Table 1.

**Table 1:** The data obtained from linearity verification.

Number of concentration level	Dilution	Theoretical concentration, µg/g (mg/L)	Measured concentration, µg/g (mg/L)	Absolute bias	Relative bias
1	L	20.82 (0.04)	20.82 (0.04)	0	0
2	0.8 L + 0.2H	430.70 (0.86)	476.05 (0.95)	0.11	10.5
3	0.6L + 0.4H	840.58 (1.68)	923.59 (1.85)	0.10	9.90
4	0.4L + 0.6H	1,250.46 (2.50)	1,285.04 (2.57)	0.03	2.80
5	0.2L + 0.8H	1,660.33 (3.32)	1,634.24 (3.27)	−0.02	−1.60
6	H	2,070.21 (4.14)	2,070.21 (4.14)	0	0

L, sample with low concentration of calprotectin. H, sample with high concentration of calprotectin.

### Verification of carry-over

The mean calprotectin concentration in the H sample was 1,624.86 μg/g (3.25 mg/L) and in the L sample was 42.65 μg/g (0.09 mg/L). The calculated carryover was 0.1 %, which does meet predefined acceptance criteria <1 %.

### Hook effect

**Figure 3:**
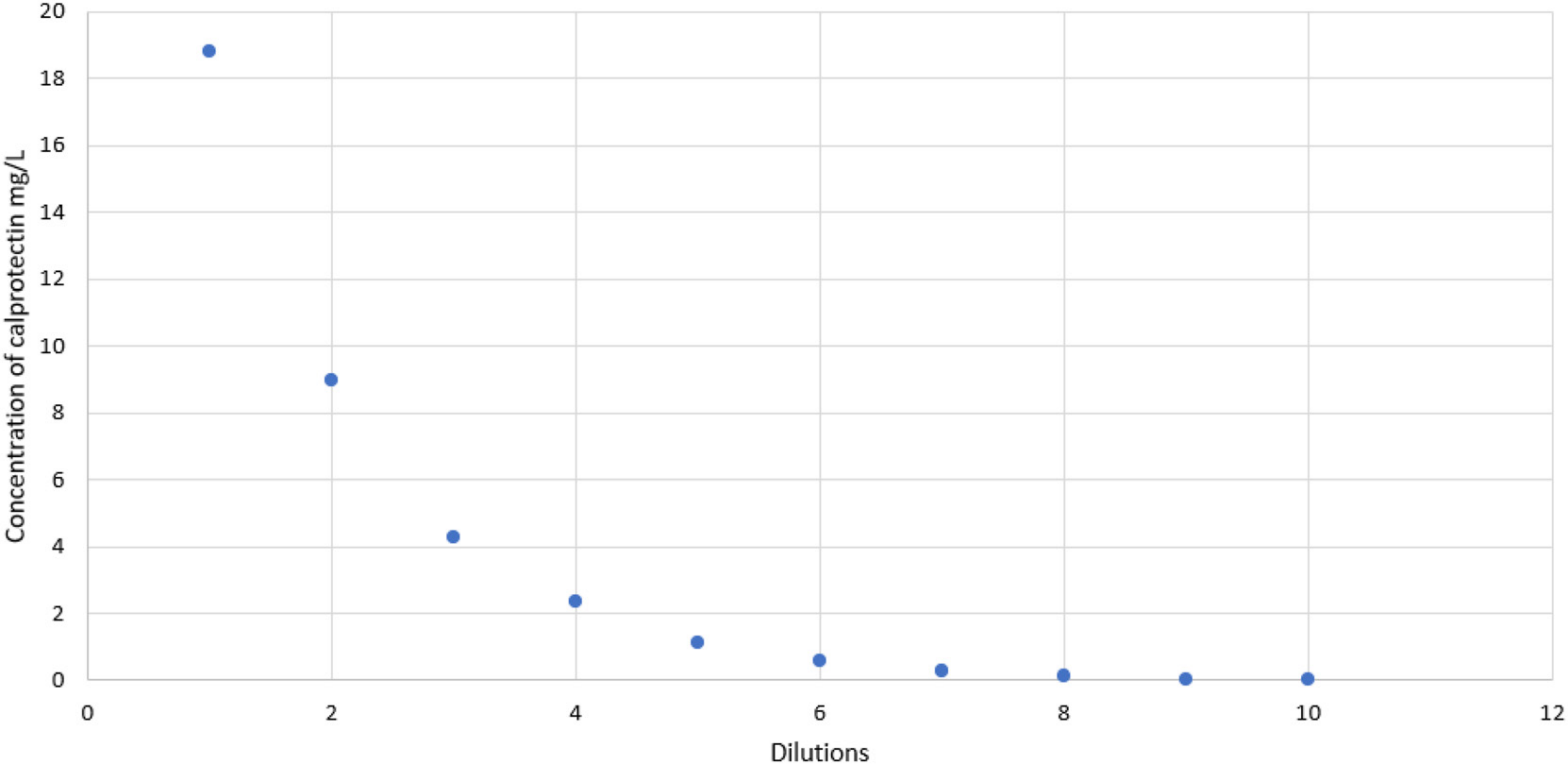
Hook effect display in graphical form. The y-axis shows the concentration of calprotectin, and the x-axis shows the number of sample dilutions.

The measured concentration of the spiked sample was 9,413.51 μg/g (18.83 mg/L). The highest level of used calibrator, the fCAL turbo Calibrator Kit (Buhlmann Laboratories AG, Schonenbuch, Switzerland) (lot 2915) was 2285.7 μg/g. All results received from the serial dilution of the spiked sample were not higher than the spiked sample results. There was no observed hook effect for determining the concentration of calprotectin in serum samples (Figure 3).

### Verification of reference intervals (RI)

In this study, 20 leftover serums were included from outpatients who met inclusion and exclusion criteria. The mean age of patients was 45 years old, with a minimum of 34 years and a maximum of 57 years. Most of the patients included were female (15/20). The proposed RI in the literature (0.68–5.45 mg/L) was verified because 20 out of 20 results were within the RI.

## Discussion

Our verification study demonstrates acceptable precision, LoB, LoQ, linearity, and the absence of the hook effect and carryover. Although, one out of six concentration levels for linearity exceeds the predefined criterion by only 0.5 %, or in absolute bias, 0.11 mg/L, which is not clinically significant. Also, the proposed literature for RI for serum calprotectin obtained by fCAL turbo Buhlmann reagent was verified. A significant proportional bias was detected when the fCAL turbo Buhlmann reagent and GCAL Gentian reagent were compared.

On one hand, there are some studies whose results are in agreement with our findings. In the study by Asberg A et al., calprotectin was measured in plasma using the same reagent as we had used in this study (Buhlmann fCAL turbo). The authors demonstrated that the method was linear, and the calculated CV % for precision was <4 % which is similar to our findings [2].

On the other hand, our findings are not in agreement with results from other published studies. The study by Nilsen T et al., was comparing Gentian GCAL reagent with Buhlmann MRP8/14 ELISA kit which is produced by the same manufacturer of one of the used reagents in our study (fCAL turbo). Unfortunately, the Passing-Bablok regression equation was shown without associated 95 % Cl where it is impossible to conclude whether there is a presence of constant or proportional bias. Consequently, it cannot be concluded whether this finding is in agreement with our results. Interestingly, Nilsen T et al. concluded that the Gentian Calprotectin immunoassay was comparable and well correlated with the Buhlmann MRP8/14 ELISA method despite that these reagents are based on different methodologies. Contrary to that, our results showed that the GCAL Gentian and Buhlamnn fCAL turbo reagents were not comparable because of the presence of proportional bias. Despite similar findings, the discrepancy between the results of all previously mentioned studies may be because of the methodology differences of used tests.

One of the limitations of this study is that more samples could have been included for the method comparison to have a wider concentration range, and the linearity should be investigated with measurements in duplicate. Although Asberg et al. stated that there is no difference of RI between gender and age, another limitation of this study was the disproportion between male and female participants in RI verification. Also, the RI could have also been verified on GCAL Gentian reagent. Lastly, the presence of carry-over should be investigated again with different and possibly more serum samples. A more extensive verification, including more samples and different reagents that have recently been introduced to the market could be investigated in future studies.

Interestingly, the values of verified RI were higher than the stated linearity by the manufacturer for the used reagent in this study, which could be considered good information to the manufacturers for the future potential development of new reagents for serum calprotectin. Moreover, it would be desirable to examine the analytical performance of the recently introduced reagent for serum calprotectin presented by the manufacturer Buhlmann whose fCAL turbo reagent was used in this study. Furthermore, additional verification of various methods for determining calprotectin is more than welcome due to the absence of international standards causing the use of various internal standards by manufacturers and therefore obstructs the better development of the reagents in terms of reducing the differences between them.

Despite the promising use of serum calprotectin as a new biomarker for diagnosing and monitoring certain diseases and investigating its clinical use and meaning, there is still a need to verify available reagents for determining calprotectin concentration. As the new reagents for the determination of calprotectin concentration are introduced on the market, they should be verified by laboratory personnel before being implemented into routine in order to have reliable and accurate results in laboratory reports.

## Conclusions

In conclusion, results obtained by verification of precision, LoB, LoQ, linearity, carry-over, and hook effect of the Buhlmann reagent meet most of the manufacturer claims in the package insert and predefined criteria for serum calprotectin, despite it being designed for the quantitative determination of calprotectin in human stool samples. The fCAL turbo Buhlmann reagent is suitable for analysing the concentration of calprotectin in serum samples. On the other hand, the fCAL turbo Buhlmann reagent is not comparable to the GCAL Gentian reagent, and the two reagents should not be used interchangeably.
